# Lack of significant association between mutations of *KCNJ10* or *FOXI1* and *SLC26A4* mutations in pendred syndrome/enlarged vestibular aqueducts

**DOI:** 10.1186/1471-2350-14-85

**Published:** 2013-08-21

**Authors:** Priya Landa, Ann-Marie Differ, Kaukab Rajput, Lucy Jenkins, Maria Bitner-Glindzicz

**Affiliations:** 1 North East Thames Regional Genetics Service Laboratory, Great Ormond Street Hospital for Children NHS Foundation Trust, 37 Queen Square,York House, London WC1N 3BH, UK; 2 Cochlear Implant Department, Great Ormond Street Hospital for Children NHS Foundation Trust, London WC1N 3JH, UK; 3 Clinical and Molecular Genetics Unit, UCL Institute of Child Health, 30 Guilford Street, London WC1N 1EH, UK

**Keywords:** Deafness, Enlarged vestibular aqueducts, Epilepsy, EVAS, *FOXI1*, Hearing impairment, *KCNJ10*, Pendred syndrome, *SLC26A4*

## Abstract

**Background:**

Pendred syndrome is a common autosomal recessive disorder causing deafness. Features include sensorineural hearing impairment, goitre, enlarged vestibular aqueducts (EVA) and occasionally Mondini dysplasia. Hearing impairment and EVA may occur in the absence of goitre or thyroid dyshormonogensis in a condition known as non-syndromic EVA. A significant number of patients with Pendred syndrome and non-syndromic EVA show only one mutation in *SLC26A4*. Two genes, *KCNJ10*, encoding an inwardly rectifying potassium channel and *FOXI1*, a transcriptional factor gene, are thought to play a role in the disease phenotypes.

**Methods:**

Using Polymerase Chain Reaction and Sanger sequencing, sixty-eight patients with monoallelic mutations of *SLC26A4* were tested for mutations in *KCNJ10* and *FOXI1*.

**Results:**

Two variants were observed in the *KCNJ10* gene, p.Arg271Cys in three patients and p.Arg18Gln in one patient; only one variant, p.Arg123Trp was observed in the *FOXI1* gene in a single patient. Both p.Arg271Cys and p.Arg18Gln are likely to be polymorphisms as judged by their frequency in the general population.

**Conclusion:**

Therefore we found no evidence for a significant association between mutations of *KCNJ10* and *FOXI1* with *SLC26A4*. It was also observed that the variant, p.Arg271Cys *in KCNJ10*, previously thought to have a protective effect against seizure susceptibility, was found in a patient with Pendred syndrome with co-existing epilepsy.

## Background

Pendred syndrome (PDS) (OMIM 274600) is one of the most common autosomal recessive disorders of hearing [1,2], characterised by mild to severe sensorineural hearing impairment (SNHL) [2-5] goitre, and inner ear malformations, typically an enlargement of the vestibular aqueduct (EVA) [6]. Some PDS cases can also exhibit Mondini dysplasia which involves the bilateral enlargement of the vestibular aqueducts (EVA) with cochlear hypoplasia [7]. EVA can also be associated with other forms of syndromic and non-syndromic deafness (DFBN4 (OMIM 600791)) [8,9], particularly non-syndromic enlarged vestibular aqueducts.

Pendred syndrome is the result of malfunction in pendrin, an apical protein anion transporter which mediates chloride (Cl^-^), hydroxide (OH^-^), bicarbonate (HCO3^-^) and iodide (I^-^) exchange [10,11]. Pendrin is expressed in the thyroid and importantly, in the inner ear to maintain the endocochlear potential [5] as well as in the kidney. Dysfunction of ion transport affects iodide organification for thyroid hormone biosynthesis [4] resulting in goitre [1,3,12], which distinguishes it from non-syndromic EVA. However, the majority of patients remain euthyroid [13]. It is now widely acknowledged that mutations in the gene *SLC26A4* located on chromosome 7q21-34 [14,15] are involved in the presentation of an EVA/PDS phenotype [14,16,17]. Biallelic mutations in the gene *SLC26A4* have been shown to cause Pendred syndrome, accounting for its autosomal recessive inheritance [9,14,18]. However, there is evidence to show that a cohort of patients affected by hearing loss and EVA, have only one mutant allele of *SCL26A4*; Yang *et al.* showed that in patients with hearing loss and EVA, in whom thyroid disorder was not used as a clinical criterion, 19% of siblings had a single mutation in *SLC26A4* and 42% had zero mutations, suggesting that other genetic factors may be involved [19] in the aetiology of their hearing loss. Rendtorff *et al.*, in a clinically well-phenotyped cohort of 109 Scandinavian patients, showed that 10% had monoallelic mutations. Likely heterogeneity of PDS and non-syndromic EVA has been proposed and investigated, with some suggesting a complex relationship between heterozygosity and the severity of the EVA/PDS phenotype [20].

Two genes, *KCNJ10* and *FOXI1*, have been investigated for their role in the PDS disease spectrum and it has been proposed that digenic mutations in both *SLC26A4* and either *FOXI1* or *KCNJ10* may cause Pendred syndrome [19,21]. The gene *KCNJ10* encodes an inwardly rectifying potassium (K^+^) channel, namely the Kir4.1 channel, expressed in a wide variety of tissues but most importantly in the case of Pendred syndrome, in the cochlear stria vascularis which maintains the endocochlear potential and K + homeostasis [22-24]. It has been shown that mice models that lacked the *Slc26a4* gene (*Slc26a4*^−/−^) did not express *Kcnj10* and that lack of this protein led to the loss of the endocochlear potential via endolymphatic acidification and Ca^2+^ absorption inhibition [25,26] which may be the direct cause of deafness in Pendred syndrome. Variants of *KCNJ10* have also been considered as a risk factor for seizure susceptibility in genetic association studies [27] and biallelic mutations in *KCNJ10* in humans are known to cause a syndromic form of hearing loss, EAST syndrome (epilepsy, ataxia, sensorineural deafness and renal tubulopathy) [28].

*FOXI1* is a transcription factor gene and an upstream regulator of *SCL26A4*[19,29]. It is also involved in the regulation of vascular H^+^-ATPase proton pumps in the inner ear, epididymis and kidney [30]. It was proposed that digenic inheritance of mutations in both *FOXI1* and *SLC26A4* were involved in the genetic basis of Pendred syndrome, as mutations in *FOX1* were shown to reduce the transcription of *SLC26A4*[19]. Moreover, *Foxi1* null mice showed phenotypic features of sensorineural deafness due to the defective pendrin-chloride mediated reabsorption in which FOXI1 was most likely a key regulator [29].

Only a few studies have found evidence that *KCNJ10* or *FOXI1* gene mutations may influence the disease phenotype shown in patients with monoallelic mutations in the *SLC26A4* gene. The present study investigates whether variants in *KCNJ10* and *FOXI1* in combination with heterozygosity for *SLC26A4* mutations are likely to be associated with the disease phenotype seen in Pendred syndrome/EVA patients (who already have one mutation in the *SLC26A4* gene). Because of the suggested role of *KCNJ10* in both epilepsy and PDS/EVA, we also screened 3 unrelated patients with the combination of Pendred syndrome and seizures.

## Methods

### Clinical and molecular evaluation of subjects

68 patient samples were referred for screening of *SLC26A4* because of a history of hearing impairment and either goitre or EVA. Available clinical details are given in Additional file 1. **All patients gave informed consent for genetic testing for diagnosis of their deafness. All were referred for genetic testing by Clinical Geneticists, Genetic Counsellors or Paediatricians.** A further 3 patients with Pendred syndrome (who had bi-allelic *SLC26A4* mutations), who had a clinical history of seizures, were also screened for specific variants in *KCNJ10/FOXI1*. In addition 95 pan-ethnic controls were sequenced for *FOXI1.*

### Genetic analyses

Genomic DNA was extracted from whole blood to standard using Flexigene (Qiagen) chemistry on the Autogen Flex (Autogen) or, where samples were received from other NHS hospitals, were extracted using their normal protocols. Polymerase chain reaction (PCR) was performed on all samples with a total reaction volume of 20 μL consisting of 0.5 μL genomic DNA, 19 μL Megamixmix (Microzone Ltd) and 0.5 μL of forward and reverse tailed primers for each gene. All 3 *KCNJ10* exons were amplified using the peltier thermal cycler (Biorad tetrad 2 DNA engine) and PCR was performed under the following PCR conditions: incubation at 95°C for 2 mins followed by 32 cycles of denaturation at 95°C for 30 seconds, annealing at 62°C for 30 seconds and an extension at 72°C for 30 seconds followed by a final 2mins at 72°C. All 4 *FOXI1* exons were amplified with the same process but with the following touchdown PCR conditions: 95°C for 2 mins followed by 95°C for 30 seconds, 65°C for 30 seconds and 72°C for 30 seconds, decreasing the annealing temperature by 1°C for a further 14 cycles then 95°C for 30 seconds, 50°C for 30 seconds and 72°C for 30 seconds for a further 19 cycles followed by a final 2 mins at 72°C.

### Primers

Primers were designed using bioinformatics software, Primer3 (v4) (http://primer3.wi.mit.edu/). No SNPs were found using SNPCheckV3 (https://ngrl.manchester.ac.uk/SNPCheckV3/snpcheck.htm;jsessionid=1D413B67748713D5A68E85D95C00D87C, NGRL Manchester).

All primers had a tailed sequence added for automated Sanger sequencing. All forward and reverse primers were tailed at the 5′ of the primer (Table 1).

**Table 1:** **Primers used to screen *KCNJ10 *and *FOXI1***

**Gene**	**Exon**	**Forward primer**	**Reverse primer**
***KCNJ10***	**2A**	GGCACATAGCAAGTGCTCAA	AGATTTCCAGGATGGTGGTG
	**2B**	TGGTGTGGTATCTGGTAGCTG	CAAAGTCACCCTCACCACTG
	**2C**	ACCCCTTACCTTCTATCATG	GTAGTATTCCTTACCAGGGC
***FOXI1***	**1A**	GCCAAGCCCTAGGGGTATAA	AGCTTCATCAGCTCCTCCTG
	**1B**	CAGGCCTATGGAGTGCAGA	CCATGCTCTCCTTCACTGCT
	**2A**	GCCTGTCGGTCTCTGTCTTC	CTCAGTCCTGGTGTGACCAA
	**2C**	CATCTTGGATGGAGCCTCAC	CCTGCTTATGTCTGGGCAGT
**Tail**		AGCTAAGCGCGAGAAGGC	AGCTAAGCGCGAGAAGGC

### Sequencing of PCR products

The complete coding regions and splice sites of the *KCNJ10* and *FOXI1* exons were subjected to bidirectional sequencing using ABI PRISM Big Dye Terminator cycle sequencing ready reaction kit (Applied Biosystems) and analysed on an ABI3730 Genetic Analyser (Applied Biosystems) using standard protocols. The data was collated using Foundation Data Collection v3.0 and were analysed using the software program Mutation Surveyor v.3.30 (Softgenetics) and in comparison to the GenBank sequences of *KCNJ10* (Genomic reference: NM_002241.4) and *FOXI1* (Genomic reference: NM_012188.4). The data was also analysed for reported variants within the gene by using Alamut (Interactive Biosoftware).

## Results

Screening of *FOXI1* and *KCNJ10* was completed for the cohort of 68 patients all of whom had previously been found to have only a single mutation in the *SLC26A4* gene. Results are shown in Table 2. In *KCNJ10,* three unrelated patients were identified with the variant p.Arg271Cys (c.811C > T) and one with p.Arg18Gln (c.52G > A). In *FOXI1*, only one patient was observed to have a variant, p.Arg123Trp (c.367C > T). This variant was not found in the 95 pan-ethnic controls. No other mutations in either *KCNJ10* or *FOXI1* were found.

**Table 2:** ***KCNJ10 *and *FOXI1 *mutations identified in patients with monoallelic mutations in *SLC26A4***

**Patient**	***SLC26A4*mutation (cDNA)**	***SLC26A4*mutation (protein)**	***FOXI1*Variant**	***KCNJ10*Variant**
25215	c.1001 + 1G > A		N	N
25278	c.1790T > C	p.Leu597Ser	N	N
34515	c.716T > A	p. Val239Asp	N	N
28979	c.1061T > C	p.Phe354Ser	N	N
35265	c.707T > C	p.Leu236Pro	N	N
40257	c.1001 + 1G > A		N	N
37952	c.1151A > G	p.Glu384Gly	N	N
38201	c.1790T > C	p.Leu597Ser	N	N
42836	c.707T > C	p.Leu236Pro	N	N
42564	(c.1234G > A	p.Val412Ile)	N	N
41066	c.1001 + 1G > A		N	N
40187	c.412G > T	p.Val138Phe	N	N
44595	c.2127delT	p.Phe709Leufs*12	N	c.811C > T (p.Arg271Cys)/N
45381	c.1790T > C	p.Leu597Ser	N	N
13343	c.1342-2_1343dup	p.Leu450Glyfs*19	N	N
45592	c.340G > A	p.Gly114Arg	N	N
48799	c.1001 + 1G > A		N	N
46182	c.707T > C	p.Leu236Pro	N	N
50939	c.2190G > T	p.Gln730His	N	N
51079	c.707T > C	p.Leu236Pro	N	N
54165	c.2T > C	p.Met1?	N	N
59858	c.2080T > C	p.Ser694Pro	N	N
61452	c.412G > T	p.Val138Phe	N	N
54483	c.1790T > C	p.Leu597Ser	N	N
63420	c.1151A > G	p.Glu384Gly	N	N
65983	c.1211C > T	p.Thr404Ile	N	N
50886	c.1151A > G	p.Glu384Gly	N	N
66609	c.-3-2A > G		N	N
66643	c.[1001 + 1G > A(;) 2219C > T]	(p.Gly740Val)	N	N
66830	c.1790T > C	p.Leu597Ser	N	N
69863	c.113T > C	p.Phe335Leu	N	N
47141	c.1790T > C	p.Leu597Ser	N	N
72446	c.-3-2A > G		N	N
72770	c.412G > T	p.Val138Phe	N	N
72617	c.1151A > G	p.Glu384Gly	N	N
76349	c.1790T > C	p.Leu597Ser	N	N
76715	c.1826T > G	p.Val609Gly	N	N
78124	c.1468A > C	p.Ile490Leu	N	N
78231	c.1001 + 1G > A		N	N
23853	c.2T > C	p.Met1?	N	N
71753	c.1342-2_1343dup	p.Leu450Glyfs*19	N	N
72950	c.1229C > T	p.Thr410Met	N	N
79945	(c.2219C > T)	(p.Gly740Val)	c.367C > T(p.Arg123Trp)	N
80435	c.707T > C	p.Leu236Pro	N	N
10576	c.[1343C > T]; [1991C > T]	p.[Ser448Leu]; [Ala664Ser]	N	N
83112	c.1790T > C	p.Leu597Ser	N	N
84175	c.119delT (c.918G > A)	p.Leu40ArgfsX26	N	N
86297	c.2153G > T	p.Phe718Ser	N	c.53G > A (p.Arg18Gln)/N
86482	c.707T > C	p.Leu236Pro	N	N
85020	c.1234G > T	p.Gln421Arg	N	N
83883	c.1790T > C	p. Leu597Ser	N	N
87823	c.1790T > C	p. Leu597Ser	N	N
84236	(c.73C > T)	(p.Pro25Ser)	N	N
88933	c.1151A > G	p.Glu384Gly	N	N
89770	c.1001 + 1G > A		N	N
90473	c.1790T > C	p.Leu597Ser	N	c.811C > T (p.Arg271Cys) /N
90511	c.1003T > C	p.Phe335Leu	N	c.811C > T (p.Arg271Cys) /N
90643	(c.970A > T)	(p.Asn324Tyr)	N	N
91820	c.1790T > C	p.Leu597Ser	N	N
40013	c.1000G > T	p.Gly334Trp	N	N
89620	c.[1790T > C(;) 412G > T]	p.[Val138Phe(;) (Leu597Ser)]	N	N
94065	c.1342-2_1343dup	p.Leu450Glyfs*19	N	N
89792	c.-103T > C		N	N
94743	c.1003T > C	p.Phe335Leu	N	N
96669	c.2015G > A	p.Gly672Glu	N	N
95020	c.1363A > T	p.Ile455Phe	N	N
99311	c.626G > T	p.Gly209Val	N	N
99458	c.1334T > G	p.Leu445Trp	N	N

Unclassified variants are denoted in brackets. p.Leu597Ser may be a benign polymorphism or hypofunctional variant [39].

Results of the analysis of the three further patients referred for *SLC26A4* screening who also had a history of seizures are shown in Table 3. One patient was found to have p.Arg271Cys, and one had the variant p.Arg18Gln.

**Table 3:** **Patients with biallelic mutations of *SLC26A4 *tested for variants within the *KCNJ10 *gene**

**Patient**	***SLC26A4*allele 1**	***SLC26A4*allele 2**	***KCNJ10 variant***	**dbSNP**
66119	c.1229C > T p.Thr410Met	c.1229C > T p.Thr410Met	c.811C > T p.Arg271Cys	rs1130183
5472	c.707T > C p.Leu236Pro	c.626G > T p.Gly209Val	c.53G > A p.Arg18Gln	rs115466046
6401	c.707T > C p.Leu236Pro	c.707T > C p.Leu236Pro	N	

## Discussion

The evidence that genetic factors in addition to mutations in the coding region of the *SLC26A4* gene contribute to Pendred syndrome and non-syndromic EVA has originated from both mouse and human studies. In humans the most compelling fact is that bi-allelic *SLC26A4* mutations are found in only a minority of patients, even those with the most severe phenotype of EVA-Mondini and thyroid dysfunction or goitre. Undiscovered non-coding region mutations of *SLC26A4* are unlikely to fully explain this observation as a number of groups have performed extensive analysis of non-coding regions with little yield, although a variant in the promoter region of *SLC26A4* which binds the transcription factor, FOXI1 was noted. Furthermore, siblings with discordant phenotypes, a mono-allelic *SLC26A4* mutation and concordant *SLC26A4* haplotypes, as well as siblings with concordant phenotypes but discordant haplotypes, indicate evidence for locus heterogeneity [9].

### Studies based on mouse models

Studies in mice have suggested other genes that might influence the PDS/EVA phenotype and may act in a digenic, or additive manner. Mice lacking FOXI1 are deaf, have inner ear malformations (EVA and cochlear dysplasia) as well as renal tubular acidosis and male infertility. Furthermore these mice show no expression of SLC26A4 in the inner ear, implying that as an upstream regulator, *FoxI1* expression is necessary for the transcription of *Slc26a4*. Therefore the contribution of *FOXI1* to EVA/PDS was investigated in humans by Yang *et al.*, who found a variant in the promoter of *SLC26A4* in a consensus binding motif for FOXI1, c.-101T > C; they showed using EMSAs that binding affinity of FOXI1 to the promoter was reduced in the presence of c.-101T > C and used Luciferase reporter constructs to show that transcription from the promoter was completely abolished in the presence of this variant [19]. Further screening of the coding region of *FOXI1* itself revealed five further non-synonymous variants, p. Asp161del, p.Arg267Gln, p.Gly335Val, p.Gly258Arg, p.Gly258Glu among 372 probands with EVA in whom one or zero *SLC26A4* mutations had previously been found. All five mutations showed reduced activation of the luciferase reporter construct. Finally mice which are double heterozygotes for variants in *Slc26a4* and *Foxi1* (*Slc26a4*^+/−^, *Foxi1*^+/−^) were reported to have EVA although hearing of these mice was not commented on [19].

The potential role of the inwardly rectifying potassium channel *KCNJ10* in PDS/EVA was also suggested from studies in mice. The *Slc26a4*^−/−^ mouse is deaf and has EVA and shows loss of endocochlear potential and no expression of KCNJ10 protein in the stria vascularis. KCNJ10 is thought to be crucial in the generation and maintenance of the endocochlear potential necessary for auditory transduction. In 2 out of 89 EVA/PDS patients who were heterozygous for a single pathogenic *SLC26A4* mutation, Yang *et al.* found two heterozygous mutations in *KCNJ10* in unrelated probands, and suggested a probable digenic interaction between these two genes. In their hands, expression of mutant KCNJ10 in Xenopus oocytes showed markedly reduced K^+^ conductance [21].

### Studies in humans

However subsequent work by others has shown that recessive mutations in *KCNJ10* do indeed cause hearing loss, but as part of a complex syndrome, EAST syndrome, consisting of Epilepsy, Ataxia, Sensorineural deafness and renal Tubulopathy. Heterozygote parents are unaffected. In addition *KCNJ10* has also been considered as a ‘susceptibility gene’ for epilepsy in humans. However the variant p.Arg271Cys was thought to be a *protective* allele against focal or generalised epilepsy [22,31,32] and yet this was found in one of our patients with co-existing seizures and Pendred syndrome. As studies failed to show a functional or structural effect on channels, and because this variant is present in 563 of 8037 individuals (0.07) of European American descent in Exome Variant Server (http://evs.gs.washington.edu/EVS/), p.Arg271Cys is most likely to be a polymorphism, unrelated to either EVA/PDS or even epilepsy. Similarly, the variant p.Arg18Gln has also been reported previously, but in a single patient with autistic spectrum disorder, seizures and intellectual disability but no deafness [33]. This suggests that it may be unrelated to any of these phenotypes, consistent with its frequency in the population (present in 1 in 57 European Americans in EVS (148 in 8452 European Americans (0.0175)) (http://evs.gs.washington.edu/EVS/). As the variant in *FOXI1*, p.Arg123Trp (c.367C > T) has not been found at a high frequency previously or in controls screened here, we cannot be sure whether this is, or is not, a pathogenic mutation. However it appears that mutations in *FOXI1* are not a major contributor to digenic inheritance in these cases.

Other genetic studies of patients with EVA/PDS have failed to find convincing evidence that mutations of *FOXI1* and *KCNJ10* contribute to these phenotypes. Chen *et al.*[34] screened *SLC26A4*, *FOXI1* and *KCNJ10* (as well as *GJB2*) in patients with bilateral deafness and inner ear malformations and found no mutations in *FOXI1* or *KCNJ10* in the 15 who had one or zero *SLC26A4* mutations; Mercer *et al.*[35] screened 51 patients with EVA and found no mutations in *FOXI1* or *KCNJ10*; Jonard *et al.*[36] screened 25 patients with unilateral deafness and unilateral EVA but found no mutations in either *KCNJ10* or *FOXI1* and Wu *et al.* screened *FOXI1* in 100 patients with EVA and found no mutations, although *KCNJ10* was not screened at that time [37]. Cirello *et al.* did however find a novel missense variant in *FOXI1* p.Pro239Leu, and 3 patients with the variant p.Arg271Cys in *KCNJ10* out of 19 patients with PDS/EVA, but functional analysis of p.Pro239Leu showed no significant impairment in the transcriptional activation of *SLC26A4,* and, p.Arg271Cys was classified as polymorphism [38].

Our study is the largest study to screen both *KCNJ10* and *FOXI1* in patients who were heterozygous for a single *SLC26A4* mutation in whom the promoter variant/*FOXI1* (c.-101T > C) binding site had already been excluded. Our results indicate that heterozygosity for mutations in either of these genes in combination with a heterozygous *SLC26A4* mutation is not a significant contributor to PDS/EVA in our cohort, even though previous *in vitro* work and mouse studies suggest their involvement in these phenotypes. The best evidence for a possible functional role of either *KCNJ10* or *FOXI1* in the PDS/EVA phenotype in humans would be genetic evidence - further families with mutations, which are shown to have a functional effect and which are present in a lower frequency in the general population than those with the PDS/EVA phenotype. Hopefully, NGS data from large sequencing efforts such as 1000 Genomes and EVS will be helpful in this respect.

It is possible that some of the cases presented here could be ‘co-incidental’ carriers for mutations in *SLC26A4* which do not contribute to their phenotype of hearing loss, or that we could have missed single or multiple exon deletions. However we think that this is unlikely to account for a significant proportion of these cases. Renorff *et al.* found no deletions in their series of patients suggesting that deletions account for a very small number of mutations in *SLC26A4*[39]. Regarding carrier ship, we have recently performed an audit of all requests for *SLC26A4* screening performed in our laboratory from 2002 onwards. This shows that where testing of *SLC26A4* was requested, 66 out of a total of 538 patients tested, had monoallelic mutations (ie. 1 in 8). Even if those with variants of unknown significance are excluded, the heterozygote carriership rate for *SLC26A4* mutations would be almost 4 times that for *GJB2,* the commonest form of inherited deafness [40]. This suggests that heterozygosity for *SLC26A4* mutations is important in the aetiology of the hearing loss in these patients.

## Conclusion

This study combined with others, suggests that variants in *KCNJ10* and *FOXI1* are not a major contributor to PDS/EVA and that screening of these genes in a diagnostic setting will add little information to genetic aetiology. Other factors, genetic or environmental, are likely to play a greater role in the aetiology of PDS/EVA.

## Competing interests

The authors declare no conflict of interest.

## Authors’ contributions

MBG and AMD designed the study and chose the patient cohort. PL performed the laboratory work under the supervision of AMD. MBG, AMD and PL all participated in the analysis of the results. PL wrote the initial manuscript which was edited and revised by MBG and AMD. KR and LJ provided critical feedback and thoughts on the manuscript. All authors read and approved the final manuscript.

## Pre-publication history

The pre-publication history for this paper can be accessed here:

http://www.biomedcentral.com/1471-2350/14/85/prepub

## Supplementary Material

Additional file 1 Clinical Information on patients. Click here for file
