# Supplementary material for: Lack of significant association between mutations of KCNJ10 or FOXI1 and SLC26A4 mutations in pendred syndrome/enlarged vestibular aqueducts
Source: BMC Med Genet. 2013 Aug 21;14:85. doi: 10.1186/1471-2350-14-85 (PMC3765178; doi:10.1186/1471-2350-14-85)
Supplement: Additional file 1 — Clinical Information on patients. [file 1471-2350-14-85-S1.docx]

**Clinical Information on patients.**

Unclassified variants are denoted in brackets. EVA is enlarged vestibular aqueducts

- Indicates no information. Cauc means Caucasian.

| **Patient** | ***SLC26A4* Mutation (cDNA)** | ***SLC26A4* Mutation (protein)** | **Hearing Loss** | **Imaging** | **Thyroid function tests TFT (age at test)** | **Perchlorate** | **Family History** | **Goitre** | **Ethnicity** |
| --- | --- | --- | --- | --- | --- | --- | --- | --- | --- |
| 25215 | c.1001+1G>A | - | - | - | - | - | - | - | Cauc |
| 25278 | c.1790T>C | p.Leu597Ser | Progressive | Normal | Normal (11) | - | No | No | Cauc |
| 34515 | c.716T>A | p. Val239Asp | Progressive | Bilat. EVA | Normal | - | Sibling affected. Not tested | No | Arab |
| 28979 | c.1061T>C | p.Phe354Ser | Progressive | Normal | Normal | - | Sister with unilat HL. Not tested | No | Cauc |
| 35265 | c.707T>C | p.Leu236Pro | Bilat. Hearing loss | Bilat. EVA | Normal (2) | - | Mild high freq HL in father. | No | Cauc |
| 40257 | c.1001+1G>A |  | Progressive | Bilat. EVA | Hypothyroid | - | No | Yes (autoimmune) | Cauc |
| 37952 | c.1151A>G | p.Glu384Gly | Bilat. Hearing loss | Bilat. EVA | - | - | 1 sib hypothyroid; 1 sib hearing loss | - | Cauc |
|  |  |  |  |  |  |  |  |  |  |
| **Patient** | ***SLC26A4* Mutation (cDNA)** | ***SLC26A4* Mutation (protein)** | **Hearing Loss** | **Imaging** | **Thyroid function tests TFT (age)** | **Perchlorate** | **Family History** | **Goitre** | **Ethnicity** |
| 38201 | c.1790T>C | p.Leu597Ser | Bilat. Hearing loss | Unilat. EVA | Normal (5) | - | Consanguinity | No | Pakistani |
| 42836 | c.707T>C | p.Leu236Pro | Progressive | Bilat. EVA | - | - | - | - | Cauc |
| 42564 | (c.1234G>A | p.Val412Ile) | - | - | - | - | - | - | Arab |
| 41066 | c.1001+1G>A |  | Bilat. Hearing loss | Bilat EVA | Normal (32) | Normal | No | No | Cauc |
| 40187 | c.412G>T | p.Val138Phe | Bilat. Hearing loss | Bilat. EVA | Normal (39) | Normal | No | No | Cauc |
| 44595 | c.2127delT | p.Phe709Leufs*12 | Unilat. SNHL | Unilat. EVA | - | - | - | - | Cauc |
| 45381 | c.1790T>C | p.Leu597Ser | Bilat. Hearing loss | Normal | Normal (35) | - | No | No | Turkish |
| 13343 | c.1342-2_1343dup | p.Leu450Glyfs*19 | Bilat. Hearing loss | Bilat. EVA | Normal | Normal | Son affected- no mutation | Yes | Cauc |
|  |  |  |  |  |  |  |  |  |  |
| 45592 | c.340G>A | p.Gly114Arg | Bilat. Hearing loss | Bilat. EVA | Normal (20) | Normal | Cousin (no mutation) | No | Cauc |
| 48799 | c.1001+1G>A |  | Bilat. Hearing loss | Bilat. EVA | - | - | - | - | Cauc |
| 46182 | c.707T>C | p.Leu236Pro | Bilat. Hearing loss asymmetrical | Bilat. EVA | - | - | - | - | Cauc |
| 50939 | c.2190G>T | p.Gln730His | Bilat. Hearing loss | Bilat. EVA | - | - | - | - | - |
| 51079 | c.707T>C | p.Leu236Pro | - | - | - | - | - | - | - |
| 54165 | c.2T>C | p.Met1? | Bilat. Hearing loss | Bilat. EVA | - | - | - | - | Cauc |
| **Patient** | ***SLC26A4* Mutation (cDNA)** | ***SLC26A4* Mutation (protein)** | **Hearing Loss** | **Imaging** | **Thyroid function tests TFT (age)** | **Perchlorate** | **Family History** | **Goitre** | **Ethnicity** |
| 59858 | c.2080T>C | p.Ser694Pro | Bilat. Hearing loss | Bilat. EVA | - | - | - | - | African |
| 61452 | c.412G>T | p.Val138Phe | Bilat. Hearing loss | Bilat. EVA | - | - | - | - | Cauc |
| 54483 | c.1790T>C | p.Leu597Ser | Unilat. Hearing loss | Unilat. EVA | Normal (43) | Normal | Daughter (no mutation) | Small autoimmune | Cauc |
| 63420 | c.1151A>G | p.Glu384Gly | Bilat. Hearing loss | - | Raised TSH | - | - |  | - |
| 65983 | c.1211C>T | p.Thr404Ile | - | - | - | - | - | - | - |
|  |  |  |  |  |  |  |  |  |  |
| 50886 | c.1151A>G | p.Glu384Gly | Bilat. Hearing loss | - | - | - | - | - | Cauc |
| 66609 | c.-3-2A>G |  | Progressive | Bilat. EVA | - | - | - | - | Cauc |
| 66643 | c.[1001+1G>A(;)  2219C>T] | (p.Gly740Val) | Bilat. Hearing loss |  |  |  |  |  | Cauc |
| 66830 | c.1790T>C | p.Leu597Ser | Unilat. Hearing loss | Unilat. EVA | Normal (1) | - | - | - | Cauc |
| 69863 | c.113T>C | p.Phe335Leu | Bilat. Hearing loss | - | - | - | - | - | Cauc |
| 47141 | c.1790T>C | p.Leu597Ser | Unilat. Hearing loss | Unilat. EVA  check | - | - | No | No | Cauc |
| 72446 | c.-3-2A>G |  | Bilat. Hearing loss | Bilat. EVA | - | - | - | - | Cauc |
| 72770 | c.412G>T | p.Val138Phe | Bilat. Hearing loss | Bilat. EVA | - | - | - | - | Cauc |
| 72617 | c.1151A>G | p.Glu384Gly | Unilat. Hearing loss | Bilat. EVA | - | - | - | - | Cauc |
| **Patient** | ***SLC26A4* Mutation (cDNA)** | ***SLC26A4* Mutation (protein)** | **Hearing Loss** | **Imaging** | **Thyroid function tests TFT (age)** | **Perchlorate** | **Family History** | **Goitre** | **Ethnicity** |
| 76349 | c.1790T>C | p.Leu597Ser | Bilat. Hearing loss | - | - | - | - | - | Cauc |
| 76715 | c.1826T>G | p.Val609Gly | Progressive | Bilat. EVA | Normal (4) | - | No | No | Cauc/African  American |
| 78124 | c.1468A>C | p.Ile490Leu | Unilat. Hearing loss | Unilat. EVA | - | - | - | - | Cauc |
| 78231 | c.1001+1G>A |  | Bilat. Hearing loss | Bilat. EVA | - | - | - | - | Cauc |
| 23853 | c.2T>C | p.Met1? | Bilat. Hearing loss | Bilat. EVA | Normal (12) | - | No | No | Cauc |
| 71753 | c.1342-2_1343dup | p.Leu450Glyfs*19 | Bilat. Hearing loss | Bilat. EVA | - | - | - | - | Cauc |
| 72950 | c.1229C>T | p.Thr410Met | Bilat. Hearing loss | Bilat. EVA | - | - | Consanguinity | - | Asian |
| 79945 | (c.2219C>T) | (p.Gly740Val) | Progressive | Bilat. EVA | - | - | - | - | ?Asian |
| 80435 | c.707T>C | p.Leu236Pro | Bilat. Hearing loss | - | - | - | Mother has Pendred (Bilat. Hearing loss, goitre and biallelic mutations) | - | Cauc |
| 10576 | c.[1343C>T];  [1991C>T] | p.[Ser448Leu];  [Ala664Ser] | Bilat. Hearing loss | Bilat. EVA | Normal (18) | - | No | No | Cauc |
| 83112 | c.1790T>C | p.Leu597Ser | Bilat. Hearing loss | Borderline enlarged EVA | Normal (1) | - | None | No | Cauc/  Ashkenazi |
| **Patient** | ***SLC26A4* Mutation (cDNA)** | ***SLC26A4* Mutation (protein)** | **Hearing Loss** | **Imaging** | **Thyroid function tests TFT (age)** | **Perchlorate** | **Family History** | **Goitre** | **Ethnicity** |
| 84175 | c.119delT  (c.918G>A) | p.Leu40ArgfsX26 | Bilat. Hearing loss | - | - | - | - | Yes | - |
| 86297 | c.2153G>T | p.Phe718Ser | Bilat. Hearing loss | Bilat. EVA | - | - | - | - | Cauc |
| 86482 | c.707T>C | p.Leu236Pro | Bilat. Hearing loss | - | - | - | - | - | Cauc |
| 85020 | c.1234G>T | p.Gln421Arg | Bilat. Hearing loss | - | - | - | - | - | Cauc |
| 83883 | c.1790T>C | p. Leu597Ser | Bilat. Hearing loss | Borderline enlarged EVA | Normal (16) | - | No | No | Asian |
| 87823 | c.1790T>C | p. Leu597Ser | Bilat. Hearing loss | Bilat. EVA | - | - | - | - | - |
| 84236 | (c.73C>T) | (p.Pro25Ser) | Unilat. Hearing loss | Bilat. EVA | Normal (1) | - | No | No | Cauc |
| 88933 | c.1151A>G | p.Glu384Gly | Bilat. Hearing loss | Bilat. EVA | - | - | - | - | Cauc |
| 89770 | c.1001+1G>A |  | Bilat. Hearing loss | - | - | - | - | - | Cauc |
| 90473 | c.1790T>C | p.Leu597Ser | Bilat. Hearing loss | Unilat. EVA | - | - | - | - | Cauc |
| 90511 | c.1003T>C | p.Phe335Leu | Bilat. Hearing loss | Bilat. EVA | - | - | - | - | Cauc |
| 90643 | (c.970A>T) | (p.Asn324Tyr) | Bilat. Hearing loss | - | Hypothyroid | - | - | - | - |
|  |  |  |  |  |  |  |  |  |  |
|  |  |  |  |  |  |  |  |  |  |
| **Patient** | ***SLC26A4* Mutation (cDNA)** | ***SLC26A4* Mutation (protein)** | **Hearing Loss** | **Imaging** | **Thyroid function tests TFT (age)** | **Perchlorate** | **Family History** | **Goitre** | **Ethnicity** |
| 91820 | c.1790T>C | p.Leu597Ser | Bilat. Hearing loss |  | Hypothyroid | - | Family history of hypothyroidism | - | Cauc |
| 40013 | c.1000G>T | p.Gly334Trp | Bilat. Hearing loss | Bilat. EVA | - | - | - | - | Cauc |
| 89620 | c.[1790T>C(;) 412G>T] | p.[Val138Phe(;) (Leu597Ser)] | Progressive | Bilat. EVA | - | - | - | - | Cauc |
| 94065 | c.1342-2_1343dup | p.Leu450Glyfs*19 | Progressive | Bilat. EVA | - | - | - | - | Cauc |
| 89792 | c.-103T>C |  | - | - | - | - | - | - | Cauc |
| 94743 | c.1003T>C | p.Phe335Leu | Bilat. Hearing loss | Bilat. EVA | Raised TSH | - | - | - | Cauc |
| 96669 | c.2015G>A | p.Gly672Glu | - | - | - | - | - | - | Cauc |
| 95020 | c.1363A>T | p.Ile455Phe | Unilat. Hearing loss | Unilat. EVA | Normal (16) | - | No | - | Cauc/  West Indian |
| 99311 | c.626G>T | p.Gly209Val | Progressive | Bilat. EVA | - | - | - | - | Cauc |
| 99458 | c.1334T>G | p.Leu445Trp | Bilat. Hearing loss | Bilat. EVA | Normal (6) | - | No | - | Cauc |
